# A Comparative Efficacy Study of Robotic Priming of Bilateral Approach in Stroke Rehabilitation

**DOI:** 10.3389/fneur.2021.658567

**Published:** 2021-07-12

**Authors:** Yi-chun Li, Keh-chung Lin, Chia-ling Chen, Grace Yao, Ya-ju Chang, Ya-yun Lee, Chien-ting Liu

**Affiliations:** ^1^School of Occupational Therapy, College of Medicine, National Taiwan University, Taipei, Taiwan; ^2^Division of Occupational Therapy, Department of Physical Medicine and Rehabilitation, National Taiwan University Hospital, Taipei, Taiwan; ^3^Department of Physical Medicine and Rehabilitation, Chang Gung Memorial Hospital at Linkou, Taoyuan, Taiwan; ^4^Graduate Institute of Early Intervention, College of Medicine, Chang Gung University, Taoyuan, Taiwan; ^5^Department of Psychology, National Taiwan University, Taipei, Taiwan; ^6^Neuroscience Research Center, Chang Gung Memorial Hospital, Taoyuan, Taiwan; ^7^School of Physical Therapy and Graduate Institute of Rehabilitation Science, College of Medicine, Chang Gung University, Taoyuan, Taiwan; ^8^Healthy Aging Research Center, Chang Gung University, Taoyuan, Taiwan; ^9^School and Graduate Institute of Physical Therapy, College of Medicine, National Taiwan University, Taipei, Taiwan; ^10^Department of Physical Medicine and Rehabilitation, Taipei Tzu Chi Hospital, Buddhist Tzu Chi Medical Foundation, Taipei, Taiwan

**Keywords:** priming, mirror therapy, bilateral upper limb training, mirror visual feedback, motor learning, stroke

## Abstract

**Background:** Stroke survivors can remain impaired in body functions, activity, and participation. A novel rehabilitation regimen is required to obtain scientific evidence and to help clinicians determine effective interventions for stroke. Mirror therapy (MT) and bilateral upper limb training (BULT) are based on the tenet of bilateral movement practice; however, the additional effect of bilateral robotic priming combined with these two therapies is unclear.

**Objectives:** This study examined the effects of two hybrid therapies, robotic priming combined with MT and robotic priming combined with BULT, in stroke survivors.

**Methodology:** The study randomized 31 participants to groups that received robotic priming combined with MT (*n* = 15) or robotic priming combined with BULT (*n* = 16). Outcome measures included the Fugl–Meyer Assessment (FMA), the revised Nottingham Sensory Assessment (rNSA), the Chedoke Arm and Hand Activity Inventory (CAHAI), and accelerometer data.

**Results:** Both groups showed statistically significant within-group improvements in most outcome measures. Significant between-group differences and medium-to-large effect sizes were found in favor of the group that received robotic priming combined with MT based on the FMA distal part subscale scores, FMA total scores, and accelerometer data.

**Conclusion:** Robotic priming combined with MT may have beneficial effects for patients in the improvements of overall and distal arm motor impairment as well as affected arm use in real life. Additional follow-up, a larger sample size, and consideration of the effect of lesion location or different levels of cognitive impairment are warranted to validate our findings in future studies.

**Clinical trial registration:** www.ClinicalTrials.gov, identifier NCT03773653.

## Introduction

Rehabilitation of stroke patients is a long process that takes several months or even years. More than 30% of stroke patients admitted to the hospital remain impaired in autonomy, engagement, and fulfillment of societal roles (1). Rehabilitation methods are needed to allow individuals to continue to maximize gains in arm impairment and function more than 3 months after stroke. Priming, an implicit learning technique, can be used to prepare the brain for a more plastic response before task-based rehabilitative therapy, thereby leading to improved functional outcomes (2). Bilateral robotic priming, an extended application of robotic therapy involving bimanual, repetitive, mirror-symmetric movement practice, is a type of movement-based priming with a low-tech robot device (3). It can normalize cortical inhibition, prepare the brain for subsequent rehabilitative therapies, and facilitate recovery through a task-oriented approach (2, 4, 5).

In recent years, reports on task-oriented approaches have increased. Mirror therapy (MT) and bilateral upper limb training (BULT) are bimanual strategies for stroke recovery and can be applied as task-oriented approaches (3, 6). BULT is performed intensively and simultaneously with both arms in a symmetrical or alternating pattern. According to the classical definition, BULT is typically symmetrical, both temporally and spatially, and can exploit the coupling effect of both arms to improve movement of the affected arm, for example, in simultaneously lifting two soft drink bottles (3, 7–11). Asymmetrical movement with different temporal and spatial relationships for the achievement of common goals, such as opening a jar of coffee or drying one's own back with a towel, has also been viewed as a kind of BULT in recent studies. BULT focuses on facilitating the coordination of a variety of different real-world tasks (12–14). For comprehensive effects, rehabilitation regimens should include not only classical definitive bilateral arm training but also the bilateral synergy framework.

MT is a promising approach in which a mirror is positioned vertically between the two arms so that the reflected image of the less affected arm gives the appearance of normal movement in the affected arm (15). The possible mechanism for the success of this therapy is that it could induce primary motor cortex cortical activations (16). Compared with BULT, MT has been proposed to provide significant benefits to distal hand function and superior improvements in sensory deficits, quality of life, and the amount of use of the affected arm (6, 17). A previous study found that BULT integrated with bilateral robotic priming was more effective than unilateral hybrid therapy for improving motor function. The efficacy was believed to result from inter limb coupling and the priming effects of bilateral symmetric practice (18). MT and BULT are both bimanual strategies for stroke; however, the distinct effect of bilateral robotic priming combined with MT and BULT is unclear.

In summary, bilateral robotic priming, MT, and BULT have been considered to be types of bilateral approaches (3, 6) and are based on the tenet of bilateral movement practice. MT and BULT can be provided as task-oriented approaches involving both arms. When combined with bilateral robotic priming, the effects of MT and BULT may be increased and differentiated. Bilateral hybrid therapy (bilateral robotic priming plus BULT) yielded a better effect on motor improvement (18). However, if bilateral robotic priming is followed by MT, which is also a type of bilateral approach but involves mirror visual feedback, the regimen may enhance the recovery effect.

This study compared the efficacy of these two different hybrid approaches that are both based on the tenet of bilateral movement practice. We hypothesized that within-group differences in the robotic primed MT (RMT) and robotic primed BULT (RBULT) groups would be found after the intervention. Furthermore, we hypothesized that sensorimotor function recovery would be better in the RMT group than in the RBULT group.

## Materials and Methods

### Research Design

This was a single-blind randomized controlled trial. An independent research assistant performed randomization by using a computer-generated random-sequence table with four permuted blocks stratified by the total Fugl–Meyer Assessment (FMA) upper arm pretest score (<35 or ≥35) (19) and the side containing the lesion (right or left). The ethics review board at each participating site approved the study protocol.

### Instruments

The primary outcome measure, the FMA, quantitatively measures the recovery of motor impairment and has high reliability, validity, and responsiveness in stroke patients (20, 21). Motor impairment levels were classified as severe (score 0–15), severe to moderate (16–34), moderate to mild (35–53), or mild (54–66) (19). The proximal (0–42), distal (0–24), and total (0–66) FMA–upper extremity (UE) scores were used to compare different UE elements in the current study.

The secondary outcome measures were the revised Nottingham Sensory Assessment (rNSA), the Chedoke Arm and Hand Activity Inventory (CAHAI), and accelerometry.

Based on the superior effects of MT on sensory improvement as reported in a previous study (8), we used the rNSA to assess sensory impairments (22). For this test, we measured (1) tactile sensation, including light touch, temperature, pinprick, pressure, tactile localization, and simultaneous bilateral touch; (2) proprioception; and (3) stereognosis. Higher rNSA scores indicate lower impairment.

RMT and RBULT are based on the tenet of bilateral movement practice; therefore, the CAHAI was chosen to assess the performance of the affected arm in 13 daily activities requiring bilateral arm function, such as opening a jar of coffee and drying one's own back with a towel. Higher scores indicate higher quality of performance (23).

To monitor the amount of use of the affected arm in real life, mean counts were collected from a wrist accelerometer, the triaxial wearable sensor GT3X+ or wGT3X-BT (ActiGraph Corporation, Pensacola, FL, USA), which was worn on the first 3 and the last 3 days of the intervention period, except when bathing. We used the vector magnitude average count, which is the average vector magnitude of all three axes during the scored time. In our case, the scored time was 3 days. During this period, activity that caused the acceleration signal was “counted” as activity. The accelerometer sampling rate was 100 Hz, and data were summed over 60-s epochs; this device has been suggested for use outside of the clinic (24, 25).

### Participant Selection

Patients were recruited by clinical staff or investigators from four outpatient clinics. A patient who was willing to join the current study could also contact the investigators *via* clinical staff. The Committee on Human Research approved the study. In accordance with the institutional review board approval, the experimental procedures, risks, and benefits of the study were explained to the potential participants after they were identified. Our recruitment procedures ensured the participants were not coerced to join the study. Patient privacy and data security were handled appropriately.

After the participants provided informed consent verbally and in writing, the study assessor conducted further eligibility and baseline assessments. A standard imaging method was used to confirm the stroke diagnosis. The inclusion and exclusion criteria were selected based on a review of the relevant literature in which potential factors were considered (6, 26). The inclusion criteria were (1) ≥3 months after the onset of a first-ever unilateral stroke; (2) patients aged 18–80 years old; (3) baseline FMA UE score between 16 and 53 (19, 27); (4) baseline spasticity score on the Modified Ashworth Scale of ≤ 3 (28); (5) ability to follow study instructions; (6) no serious vision, neurologic, orthopedic, or medical problems based on medical history data and physical examinations; (7) no Wernicke's aphasia in which the participant may have difficulty following the instructions for assessments and intervention; and (8) no participation in other studies.

To date, no published research has compared the effects of RMT with RBULT among stroke patients. Thus, the sample size required for this study was estimated based on a previous study (6). Based on the smallest sample size needed for achieving a statistical power of 0.80 with a one-sided type I error of 0.05, we deemed a total sample size of 28 with 14 subjects for each group was sufficient to validate the advantages of MT on somatosensory function.

### Procedures

All participants received 40–45 min of bilateral robotic priming and 40–45 min of MT or BULT. As determined from the designs of previous studies on bimanual strategies and the feasible number of clinical patient visits to the hospital (6, 18), the schedule for both groups was 3 days/week for 6 weeks. The intervention was delivered by three licensed and certified occupational therapists. To confirm that the intervention was provided as intended, practice guidelines were used, and the principal investigator supervised these sessions.

The Bi-Manu-Track robot (Reha-Stim Co., Berlin, Germany) was used for bilateral robotic priming practice. The robot enables two symmetric movements (forearm pronation/supination and wrist flexion/extension) in three treatment modes (passive–passive mode, active–passive mode, and active–active mode). A computer task was supplied with the robot practice to enhance the participant compliance. The participants performed ~11,200–1,600 repetitions of the movements each day.

**Figure 1:**
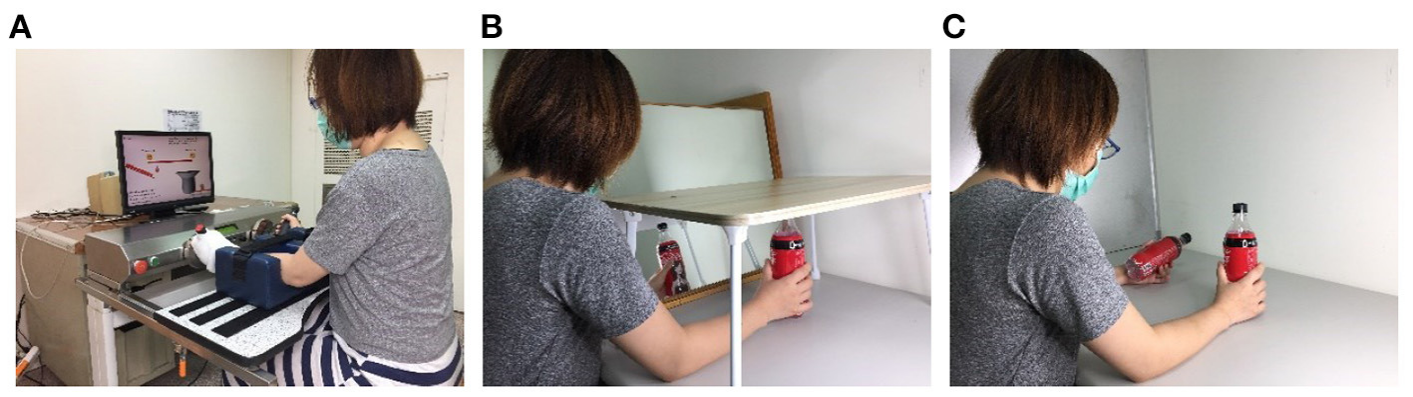
Intervention setup for Bi-Manu-Track robot **(A)**, mirror therapy **(B)**, and bilateral upper limb training **(C)**.

During MT, a wooden mirror box (41 × 50 × 33 cm^3^) was placed so that the mirrored side was in the midsagittal plane of the participant (29). The participant was then guided to watch the mirror image of the movement of the less affected arm and move both arms as symmetrically as possible. To ensure that the participants watched the mirrored image, the less affected arm was hidden by a bed tray table (30) (Figure 1). The intervention activities involved task-oriented activities such as picking up and putting down items in a box, lifting two soft drink bottles, and other functional tasks involved in daily activities.

During BULT, the participants performed different types of tasks, including (1) common goal activities that depend heavily on cooperation between both arms, such as pulling up the trousers or spooning soup out of a bowl, and (2) independent goal practice tasks in which the arms are not necessarily interdependent, such as simultaneously lifting two soft drink bottles or manipulating two coins (Figure 1) (12, 14).

In addition to the clinic-based RMT or RBULT, each participant practiced the transfer package at home for 5 days/week to transfer the improved abilities to real-life situations. The transfer package included a behavioral contract, three home skills practices (e.g., lifting two soft drink bottles, picking up and putting down items in a box, or lifting a plate with both hands) for a total of 30 min, six important daily activities involving the affected arm (e.g., taking clothes out of the closet, eating snacks, or turning on the light), and a home diary to record the amount of use of the affected arm (Figure 3). The therapists helped participants with compliance management and problem solving at each clinic visit (6, 31).

All interventions were graded and designed according to the level of impairments and life experience of the participants as well as their individual needs and rehabilitation goals (5, 6, 18, 29). To foster engagement in the practice of transfer tasks, we interviewed the participants to identify the main problem areas and set their three prioritized goals. For example, a 48-year-old housewife who sustained a left hemispheric stroke set kitchen activities as one of her prioritized goals of functional recovery. Because the participant experienced difficulty in flexing her affected arm during kitchen activities, the therapist instructed her to practice lifting a hot water kettle to make a cup of tea. As she improved in task performance, the kettle was filled with more water to make the task more challenging. The treating therapists worked with the participants to identify the skills needed for practice to ensure the transfer package fit into the personal needs of the participants as they progressed overtime. Any other routine interdisciplinary rehabilitation without emphasis on arm training continued as usual.

### Data Collection

Outcome assessments were conducted at baseline and immediately after the intervention. The fully trained assessor was a licensed occupational therapist blinded to the group assignments, and the participants were blinded to the study hypotheses.

### Data Analysis

Depending on the data type, within-group data and between-group data were analyzed using χ^2^ tests, paired *t-*tests, or independent *t-*tests. The treatment assignment in this randomized controlled trial depended on the baseline score; therefore, analysis of covariance was used to achieve higher power (32). The effect size between the two groups, eta-squared (η^2^), was calculated by analysis of covariance, and large, moderate, and small effect sizes were represented by η^2^ values of at least 0.14, 0.06, and 0.01, respectively (33). The baseline score was the covariate, the group was the independent variable, and the posttreatment score was the dependent variable. For each subscale of the rNSA, only participants who had less than a perfect score at baseline (demonstrating sensory impairment) were included in the data analysis. The significance (α) level was set at 0.05. The analyses were performed using SAS 9.4 (SAS Institute Inc., Cary, NC, USA) and G^*^Power 3.1 (Heinrich-Heine-Universität Düsseldorf, Düsseldorf, Germany) software.

## Results

**Figure 2:**
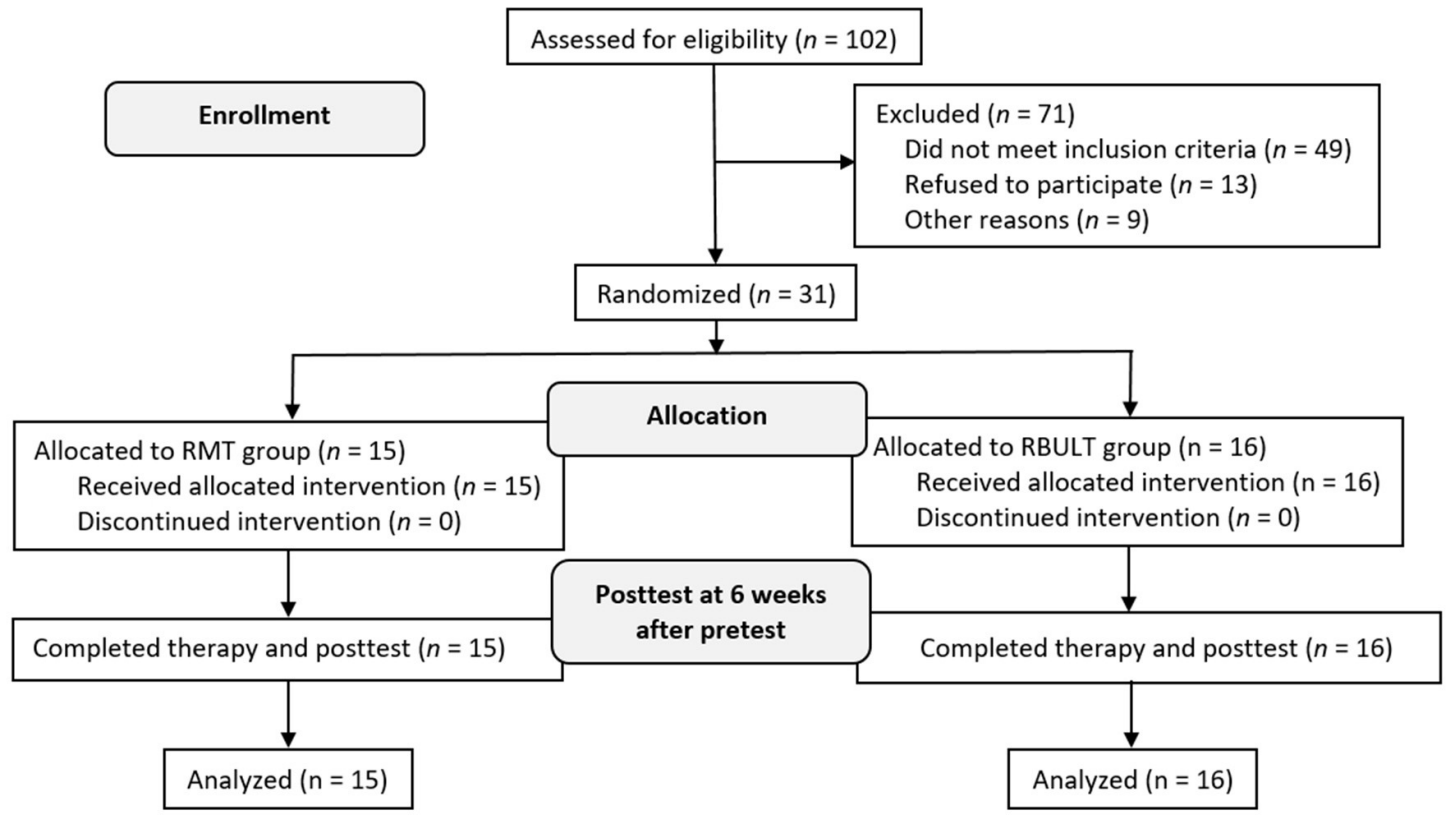
Consolidated standards of reporting trials flowchart of the study.

The study recruited 31 patients [19 men (61.29%)] who were randomly assigned to the RMT (15 patients) or RBULT (16 patients) group (Figure 2). The cohort has a mean age of 55.53 (SD, 12.16) years and a mean of 19 (SD, 16.80) months after stroke. The two treatment groups were statistically equivalent in baseline demographics and clinical characteristics, motor impairment level, and stroke severity (Table 1). All participants finished the 6-week intervention and posttreatment assessment.

**Figure 3:**
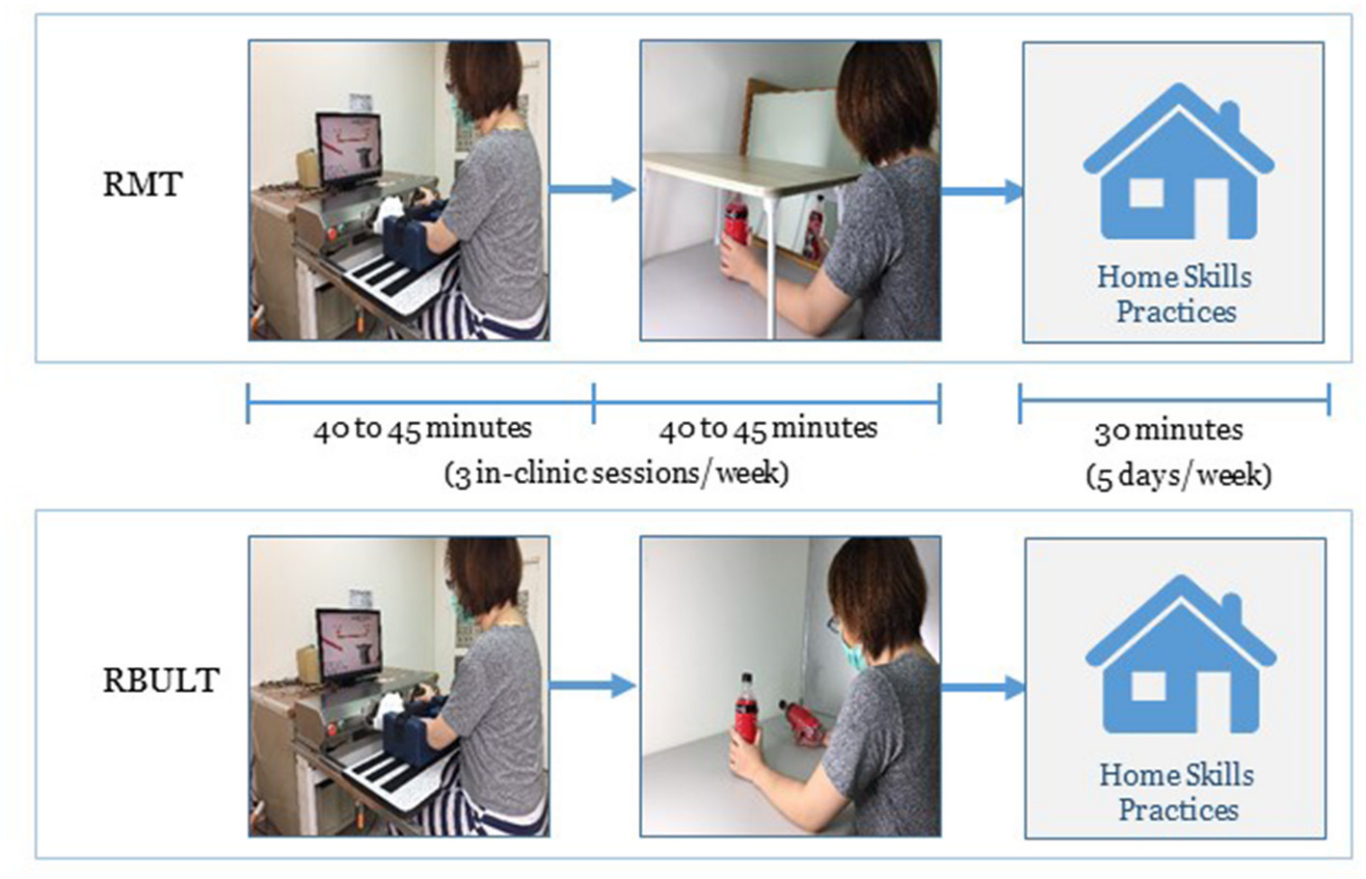
Diagram of the study design.

**Table 1:** Demographics and baseline clinical characteristics.

**Characteristics**	**RMT (*n* = 15)**	**RBULT (*n* = 16)**	***p-*value**
Age, mean (SD), years	55.71 (9.2)	55.36 (14.72)	0.94
Sex, *n* (%)			
Male	8 (53.33)	11 (68.75)	0.47
Female	7 (46.67)	5 (31.25)	
Side of lesion, *n* (%)			
Right	9 (60.00)	9 (56.25)	>0.99
Left	6 (40.00)	7 (43.75)	
Type of stroke, *n* (%)			
Hemorrhage	8 (53.33)	5 (31.25)	0.29
Ischemia	7 (46.67)	11 (68.75)	
Months after stroke, mean (SD)	18.13 (15.11)	20.00 (18.69)	0.76
FMA-UE, mean (SD)	34.93 (7.7)	32.44 (7.68)	0.37
NIHSS, mean (SD)	5.67 (2.02)	6.31 (2.89)	0.48

*FMA-UE, upper extremity motor subscale of the Fugl–Meyer Assessment; NIHSS, National Institutes of Health Stroke Scale; RBULT, robotic primed bilateral upper limb training; RMT, robotic primed mirror therapy; SD, standard deviation*.

The post treatment evaluation showed statistically significant within-group improvements in most outcome measures in both groups, including the FMA score, temperature perception, touch localization, tactile total scale score, proprioception, and the CAHAI score. Statistically significant within-group improvements in light touch perception, pinprick perception, and accelerometer data were shown only in the RMT group, whereas significant improvements in stereognosis were shown only in the RBULT group (Table 2). Moreover, statistically significant intergroup differences with medium to large effect sizes in favor of the RMT group were found in the FMA distal part subscale score (*p* = 0.03), FMA total score (*p* = 0.01), and accelerometer data (*p* = 0.02). These results reflected the distinctive effects of RMT in improving motor function and the actual amount of functional arm use.

**Table 2:** Descriptive and inferential statistics of the outcome measures at baseline and posttest per treatment group.

**Outcome measures**	**Baseline, mean (SD)**		**Post treatment, mean (SD)**		**ANCOVA**	
	**RMT (*n* = 15)**	**RBULT (*n* = 16)**	**RMT (*n* = 15)**	**RBULT (*n* = 16)**	***p*-value between groups**	**Effect size η^**2**^ (95% CI)**
**FMA-UE**						
Proximal	27.4 (4.03)	26.63 (4.57)	31.53 (3.62)^**^	29.94 (5.12)^**^	0.12	0.05 (0–0.25)
Distal	7.53 (4.53)	5.81 (4.04)	11.93 (6.8)^**^	8.5 (5.37)^**^	0.03^§^	0.07 (0–0.28)
Total	34.93 (7.7)	32.44 (7.68)	43.47 (9.43)^**^	38.44 (9.67)^**^	0.01^§^	0.13 (0–0.35)
**rNSA^a^**						
Light touch	7.86 (4.38)	8.33 (4.27)	9.71 (4.79)^*^	9.67 (4.55)	0.34	0.01 (0–0.28)
Temperature	9.82 (6.21)	9.3 (5.7)	11.91 (5.52)^**^	11 (5.62)^**^	0.29	0.02 (0–0.25)
Pinprick	8.86 (4.45)	9.83 (5)	11.71 (6.34)^*^	12.83 (4.49)	0.54	<0.01 (0–0.10)
Pressure	11.33 (5.09)	11.4 (4.93)	12.5 (5.54)	15.6 (5.37)	0.93	0.24 (0–0.55)
Localization	7.62 (4.03)	9.47 (6.46)	10.08 (4.55)^*^	11.2 (6.57)^**^	0.20	0.02 (0–0.21)
Bilateral simultaneous touch	7.33 (6.92)	4.2 (3.03)	10.83 (5.04)	7.4 (8.32)	0.47	0.01 (0–0.31)
Tactile total scale	74.86 (27.69)	79.73 (31.2)	83.14 (23.09)^**^	86.67 (24.96)^*^	0.35	<0.01 (0–0.08)
Proprioception	16.1 (5.13)	14.36 (3.98)	17.8 (4.59)^*^	15.91 (4.85)^*^	0.43	<0.01 (0–0.18)
Stereognosis	9.73 (9.13)	12.62 (7.96)	12.55 (9.15)	13.85 (8.55)^**^	0.14	0.06 (0–0.29)
**CAHAI**	39.2 (11.59)	37.25 (12.74)	51.4 (13.98)^**^	47 (13.63)^**^	0.07	0.07 (0–0.28)
**Accelerometer**	453.32 (206.37)	443.64 (188.73)	541.2 (247.22)^*^	433.38 (197.15)	0.02^§^	0.14 (0–0.36)

*Values are presented as the mean (standard deviation)*.

*CAHAI, Chedoke Arm and Hand Activity Inventory; RBULT, robotic primed bilateral upper limb training; RMT, robotic primed mirror therapy; rNSA, revised Nottingham Sensory Assessment*.

a *Only those participants who scored less than full scores at baseline, indicating sensation impairments, were included in the data analysis. The numbers of individuals in the RMT group and RBULT group were 7 vs. 6, 11 vs. 10, 7 vs. 6, 6 vs. 5, 13 vs. 15, 6 vs. 5, 14 vs. 15, 10 vs. 11, and 11 vs. 13 for the analysis of light touch, temperature, pinprick, pressure, localization, bilateral simultaneous touch, tactile total scale, proprioception, and stereognosis, respectively*.

§ *p-value < 0.05 in the between-group analysis*.

* *p-value < 0.05 in the within-group analysis*.

** *p-value < 0.01 in the within-group analysis*.

After the adaptation phase of the study, no intolerable adverse events related to treatment were reported. In light of our findings, with a one-sided type I error rate of 0.05 and an effect size (η^2^) of 0.07 to 0.14, the power of our study was 31–58%.

## Discussion

Prior studies have noted the additional effect of priming combined with a task-oriented approach. For chronic stroke patients, combining movement-based priming and task-oriented approaches can be a promising intervention strategy for recovery in the arms (34). The objective of the study was to identify the efficacy of RMT and RBULT, two different hybrid approaches that are based on the tenet of bilateral movement practice. The study results indicate that both groups of stroke survivors showed significant benefits in the recovery of most sensorimotor functions. Furthermore, RMT may significantly improve distal and total arm motor impairment as well as the actual amount of functional arm use, in accordance with previous findings. However, this study was unable to demonstrate consistent evidence of sensory recovery.

Studies have demonstrated that MT is a promising method to restore motor function in the distal arm (21, 29, 35). Superior motor recovery in the distal arm with RMT can be explained by the following factors. First, in our study, the participants in the RBULT group involuntarily watched the performance of the affected hand during therapies, whereas the use of the bed tray table in the RMT group forced participants to look at the reflected movement of the less affected arm. The forced visual perception of the arm movement of the less affected arm may contribute to motor learning and elicit increased therapy effects (36). In addition, the conflicting spatial relationship between real and reflected objects reinforces the difficulty of MT task-oriented activities. The participants in the RMT group needed to focus more intently on performing activities carefully and correctly and therefore may have achieved better performance and recovery. In addition, the participants with good proprioceptive function could directly correct their movements by comparing the visual input from the reflected image superimposed on the affected arm and the proprioceptive inputs from that arm. On the other hand, for the participants with impaired proprioceptive function, observing the action of the superimposed reflection may have guided the movements of the affected arm more directly and reduced their motor impairment. However, in the current study, the limited space available for movement under the bed tray table may also have limited the recovery of the proximal part in the RMT group, preventing any significant between-group benefit in favor of RMT for the proximal limb.

Another clinically significant finding was that the mean differences in the FMA and CAHAI scores of both groups after the study intervention were higher than the minimal clinically important differences (37–39). Compared with other recent studies with a comparable time since stroke and comparable baseline impairment as indicated by the FMA, this study showed a mean difference in the RMT group that was much larger than the minimal clinically important differences and the mean difference in robotic therapies without MT (40). Higher mean differences were also found in the comparison of the CAHAI scores between this study and our previous study (41). However, there were similar findings on the combination of robot-assisted therapy and constraint-induced therapy (42). In addition, numerous priming techniques can be combined with task-oriented approaches. Similar findings have been reported for stimulation-based and manipulation input sensory priming techniques assessed with the FMA (34, 43, 44). This may indicate the efficiency of combining contemporary therapies with priming techniques and the need to clarify the best combination regimen in future studies.

A previous MT study that used motor activity logs to investigate the self-perceived assessments of patients of their use of the affected arm found only a non-significant trend in increased arm use (6). To the best of our knowledge, this is the first study investigating the effect of MT on the objective amount of use as an outcome variable. The RMT group in our study demonstrated a significant improvement, the causes of which may include the following: first, in contrast to self-perceived assessment with a motor activity log, accelerometers can capture all activity without over- or under-reporting results due to recall bias; therefore, these devices can objectively quantify the true improvement in the amount of affected arm use after the RMT. Second, this improvement may have been the result of significant recovery of the distal arm, enhancing the participants' motivation to use their affected arms. Learned nonuse was thus reduced, and the use of the affected arm was increased.

Finally, the significant between-group effects for somatosensory function were not consistent with previous studies. Findings on the recovery of sensory function have varied across randomized controlled trials of MT. Some studies found beneficial effects on the degree of improvement in cutaneous sensitivity, temperature perception, or pain perception, whereas other studies did not find any differences (6, 45). Different MT regimens involve different active or passive sensory stimulation protocols that may have different effects on sensory recovery. These findings may need to be clarified in a well-designed study.

### Limitations and Future Research

Our study has limitations. First, the results of priming may be evident at follow-up (2, 4, 46). Without a follow-up evaluation, we could not determine whether the greater immediate effects in the RMT group were maintained. Second, the sample size was small. A minimum total sample size of 56 with 28 subjects for each group would have provided a statistical power of 0.80 with a two-sided type I error threshold of 0.05 in improving motor impairment (i.e., the FMA total score). Third, we did not consider the effect of lesion location or different levels of cognitive impairments. These factors should be taken into account in future studies.

In recent years, the age of stroke patients has a decreasing trend (47). The average age of the participants in this study was much lower than the average age of stroke patients, and age may influence activities of daily living improvement (48). In addition, the participants had moderate to mild impairment on average, and the therapy administered may be more limited or more beneficial to stroke survivors with different levels of impairment. These limitations imply that a careful interpretation of the study results is needed. Finally, combining bilateral robotic priming and task-oriented approaches to arm treatment could be a promising intervention strategy for reducing motor impairment and enhancing affected arm use. However, regarding the activity level in the International Classification of Functioning, Disability, and Health domains, for other types of priming (e.g., stimulation and sensory priming), there is no conclusive evidence to support the combined use of priming with task-oriented approaches (36). Our study may serve as basis for future studies.

## Conclusions

Stroke survivors can remain impaired at least 3 months after onset. This is the first study to compare the efficacy of robotic priming of MT and BULT for stroke; it provides scientific evidence as well as a reference for clinicians in determining effective interventions. The results suggest that both hybrid therapies provide benefits in motor improvement; however, RMT may promote better recovery in the distal parts of the arm and the entire arm as well as lead to a greater increase in the actual amount of functional arm use. In individually tailored rehabilitation therapy plans involving bilateral practice, RMT may be a better option if improvements in motor impairment or increases in the use of the affected arm are the goal of treatment. The results of this study warrant further research with a larger sample to address the retention of therapeutic benefits.

## Data Availability Statement

The datasets presented in this article are not readily available because based on the Personal Information Protection Act enacted in Taiwan, individualized data cannot be released for the protection of privacy. Requests to access the datasets should be directed to kehchunglin@ntu.edu.tw.

## Ethics Statement

The studies involving human participants were reviewed and approved by Research Ethics Committee, National Taiwan University Hospital. The patients/participants provided their written informed consent to participate in this study.

## Author Contributions

K-CL, C-LC, GY, Y-JC, and Y-YL contributed to the conception of the work. K-CL designed the experiments. Y-CL performed the experiments. K-CL and Y-CL analyzed the data and wrote the manuscript. C-TL contributed materials. All authors contributed to the article and approved the submitted version.

## Conflict of Interest

The authors declare that the research was conducted in the absence of any commercial or financial relationships that could be construed as a potential conflict of interest.
